# A study of factors influencing self‐stigma in people with epilepsy: A nationwide online questionnaire survey in Japan

**DOI:** 10.1002/epi4.12661

**Published:** 2022-10-25

**Authors:** Izumi Kuramochi, Takayuki Iwayama, Koko Oga, Takafumi Shiganami, Tomoki Umemura, Sayaka Kobayashi, Takaaki Yasuda, Haruo Yoshimasu

**Affiliations:** ^1^ Department of Psychiatry, Saitama Medical Center Saitama Medical University Saitama Japan; ^2^ Mara Hospital, Bethel Epilepsy Center Bielefeld University Bielefeld Germany; ^3^ Department of Psychology Showa Women's University Tokyo Japan; ^4^ Department of Nursing, Saitama Medical Center Saitama Medical University Saitama Japan

**Keywords:** epilepsy, patients with epilepsy (PWE), questionnaire survey, self‐esteem, self‐stigma

## Abstract

**Objective:**

Epilepsy carries a significant stigma. While there is some evidence that self‐stigma accompanies a lack of knowledge about epilepsy, there are no studies in Japan. This study aimed to determine factors contributing to self‐stigma in Japan.

**Methods:**

We conducted an online questionnaire survey. Three hundred and ten patients completed the questionnaire (mean age of 47.8 ± 11.9) in contrast to the total registered users on the questionnaire site as 28 315 from Jul 29 to Aug 2, 2021. We asked about demographic variables, satisfaction with treatment, limitations in life, support status, seizure frequency, the Epilepsy Self‐Stigma Scale (ESSS), the Rosenberg Self‐Esteem Scale (RSES), and the Epilepsy Knowledge Scale (EKS). We conducted the statistical analysis on self‐stigma, self‐esteem, knowledge of epilepsy, and seizure frequency associations by Spearman's rank correlation.

**Results:**

The mean value of the EKS was 40.19 ± 18.75, the ESSS was 17.69 ± 6.25, and the RSES was 26.02 ± 6.13. We identified a significant moderate negative correlation between self‐esteem and self‐stigma (*P* < .001, *ρ* = −.423), a significant weak positive correlation between self‐esteem and knowledge (*P* = .005, *ρ* = .177), and a significant weak negative correlation between seizure frequency and self‐stigma (*P* < .001, ρ = −.162). Of the 186 respondents who were working or studying, 91 (49%) participants had experienced any discrimination due to epilepsy. The use of psychosocial support, that is, participating in epilepsy self‐help groups and educational programs, was 5.8%.

**Significance:**

There was no correlation between the strength of self‐stigma and the knowledge, while there was a positive correlation between self‐esteem and knowledge (*P* = .005, *ρ* = .177). There was a negative and weak correlation between seizure frequency and self‐stigma (*p* < .001, *ρ* = −.162). These results suggest that sufficient knowledge may improve the self‐esteem and thus reduce the self‐stigma. In addition, short‐term treatment for seizure control is insufficient to reduce self‐stigma. The dissemination for people to enable sufficient epilepsy knowledge and positive perceptions of epilepsy by increasing self‐efficacy throughout a lifetime may reduce self‐stigma.

**Key points**

- The goal of this study was to identify the factors associated with self‐stigma that affect patients with epilepsy in Japan.
- There was no correlation between the strength of self‐stigma and knowledge of people with epilepsy, but there was a positive correlation between self‐esteem and knowledge.
- There was a negative and weak correlation between seizure frequency and self‐stigma.
- Self‐help groups and educational programs provide opportunities to gain knowledge about epilepsy, but our survey's participation rate was 5.8%.

## 1 INTRODUCTION

The stigma associated with epilepsy is prevalent in various cultures.^1^ It is frequently regarded as one of the most significant issues affecting the lives of patients with epilepsy (PWE) and their families.^2^, ^3^, ^4^, ^5^ According to a report by the International League Against Epilepsy (ILAE) Task Force on Stigma in Epilepsy in 2022,^6^ the prevalence of stigma varies significantly by region, and factors contributing to the development of stigma include a lack of understanding of epilepsy and a lack of educational opportunities.^7^ Stigma accompanies a lack of information about epilepsy, low educational achievement, low socioeconomic status, undeveloped living environments (e.g., villages, etc.), and prejudices from religious (superstitious) beliefs.

Stigma perception has been linked to increased seizure frequency, younger onset age, longer duration of epilepsy, lower educational attainment, less understanding of epilepsy, and younger age.^1^, ^2^, ^3^ A significant result is that epilepsy terminology may contribute to stigma generation. For instance, the Japanese name for epilepsy, “tenkan,” carries the negative connotation of “kuruu” (to go insane) and “a violent disposition prone to infatuation.” As a result, the name itself carries a derogatory connotation. While epilepsy is widely recognized as a disease caused by abnormal neuronal activity in the brain, stigma, and discrimination against people with epilepsy remain deeply ingrained in Japanese culture.^8^

**FIGURE 1:**
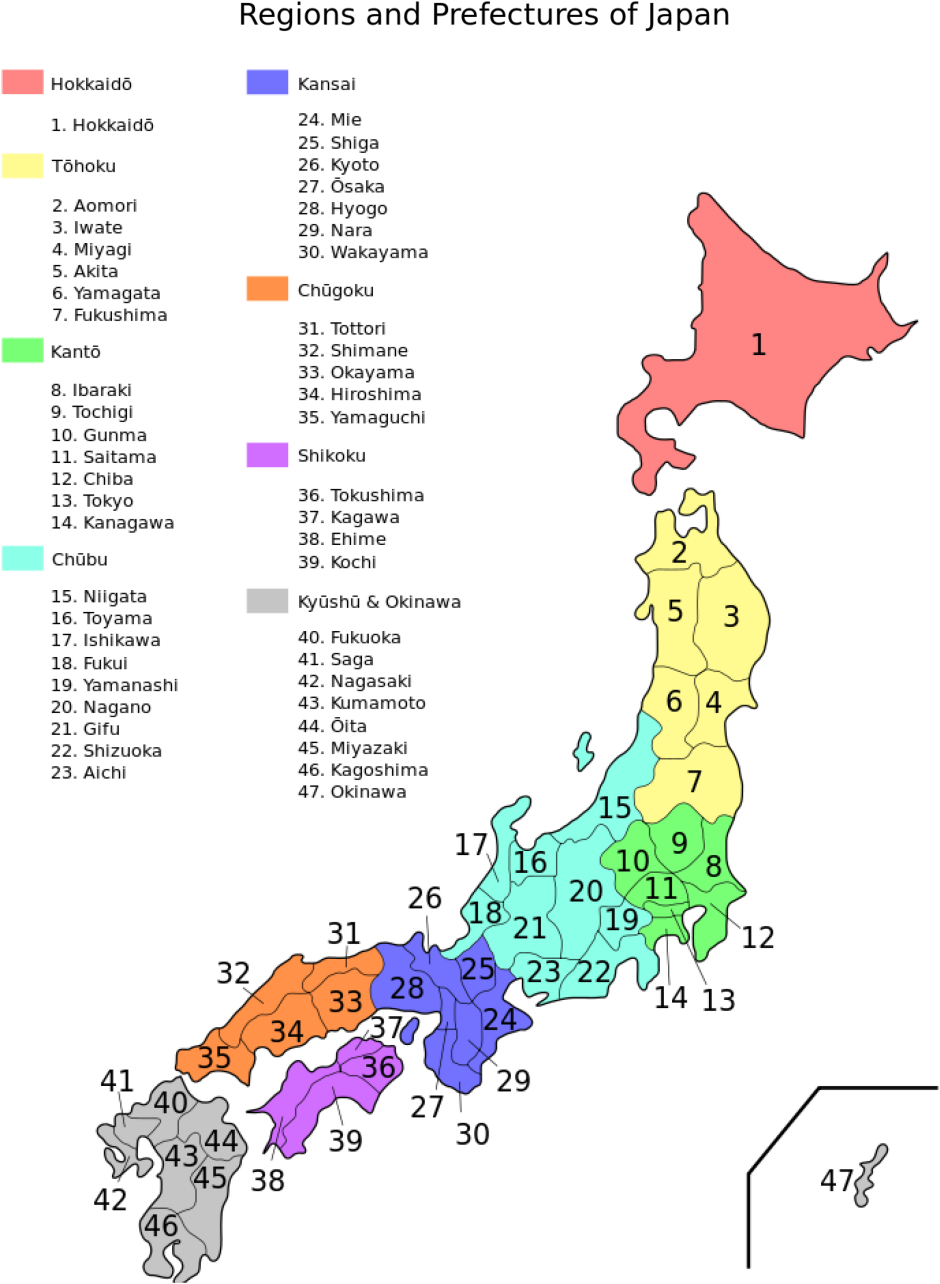
Regions and prefectures of Japan

Traditionally, geographers divide Japan into eight sections/regions (Figure 1).The population is 127 million, and the capital city of Tokyo exists in the center of the largest island in Japan, with about 40 million people. The prevalence of epilepsy in Japan is about 0.8%, and about 1 million people have epilepsy. There are 37 epilepsy centers registered with the Japan Epilepsy Center Association (12 of which are in the Kanto region, Tokyo metropolitan area). While there is no significant difference in the education or medical technology levels across Japan, most universities and medical institutions are located in the Tokyo metropolitan area.

Previous reports^7^, ^9^ have shown that the frequency of seizures, the lack of knowledge about epilepsy, the impressions and beliefs of patients, and the general population about epilepsy influence the stigma of epilepsy among patients with epilepsy. Also, according to a survey report from Turkey, education, income, age of onset, seizure frequency in the previous year, social support, and knowledge of and attitudes toward epilepsy were essential factors in determining stigma scale scores.^10^ Moreover, some previous studies have described an association between high seizure frequency and high stigma of epilepsy.^11^, ^12^, ^13^ However, the variables affecting stigma among Japanese PWE are still unknown. In a previous study, the authors devised a questionnaire to assess self‐stigma among PWE that is culturally sensitive to the Japanese.^8^, ^14^ The Epilepsy Self‐Stigma Scale (ESSS) is an eight‐item scale with subscales comprising three factors: Internalization of stigma (internalized stigma), Societal incomprehension (perception of stigma), and Confidentiality (actions taken to avoid stigma).^14^ Higher scores indicate greater self‐stigma caused by epilepsy. Additionally, the subscale scores indicate which dimensions of self‐stigma are more prevalent in PWE.

In this study, we used the questionnaire developed by the authors to ascertain perceived self‐stigma in Japan and to identify social and psychological elements contributing to PWE's stigmatization.

## 2 METHODS

### 2.1 Participants and procedure

Using an online questionnaire, we surveyed patients with epilepsy registered with an online survey service (Intage Inc.). This study collected responses from people throughout all sections of Japan. Three hundred and ten patients completed the questionnaire (mean age of 47.8 ± 11.9 years old, 190 males and 120 females) in contrast to the total registered users on the questionnaire site as 28 315 (total request numbers, including people with not epilepsy) from Jul 29, 2021, to Aug 2, 2021.

### 2.2 Measures

We requested patients to answer the questionnaire, age, gender, educational history, employment status, marital status, restrictions of daily life, the condition of support, satisfaction with current support, seizure frequency in the past 6 months, seizure types (subjective by the patient, not by the name of the diagnosis), perceived worsening of seizures, and side effects of antiseizure medications, the epilepsy self‐stigma scale (ESSS), the Rosenberg self‐esteem scale (RSES), and the epilepsy knowledge scale.

#### 2.2.1 Epilepsy self‐stigma scale (ESSS)

The ESSS is an eight‐item scale with subscales comprising three factors: Internalization of stigma (internalized stigma), Societal incomprehension (perception of stigma), and Confidentiality (actions taken to avoid stigma).^14^ The ESSS classified into 4‐point Likert‐type scales: “1: Disagree”, “2; Slightly Agree”, “3; Agree”, “4; Strongly Agree.” The total score ranges from 8 to 32 points. The higher score was interpreted as a higher self‐stigma caused by epilepsy. The total scale can be used to assess self‐stigma in PWE. Higher scores indicate greater self‐stigma caused by epilepsy. Furthermore, the subscale scores indicate which aspects of self‐stigma are the most significant/relevant in PWE. Separate assessments of 1 and 2–4 response items can also be used to assess the presence or absence of patient self‐stigma.

#### 2.2.2 Rosenberg self‐esteem scale (RSES)

Self‐stigma is negatively correlated to self‐esteem.^15^, ^16^ This study uses the RSES^17^, ^18^ to assess the extent of self‐esteem to examine the construct validity of the ESSS. Items are rated on a five‐point Likert scale from 1 (strongly disagree) to 4 (strongly agree). Total scores range from 10 to 40. Higher scores indicate higher levels of self‐esteem. We used the Japanese version of the RSES,^19^ the method identified with high validity and reliability using back translation.

#### 2.2.3 Epilepsy knowledge scale

To assess epilepsy‐specific knowledge, we used the Epilepsy Knowledge Scale developed by Theodor W. May et al.^20^ A Japanese version of this scale (18 items) was created by Inoue et al. and back‐translated and is already used in epilepsy research in Japan.^21^ This scale used three answer categories (“true,” “false,” or “I do not know”) instead of two categories since patients with epilepsy do not have to select specific answers mandatorily. This scale was created with 18 items in Japanese, but to make the evaluation easier to understand, we counted the number of questions answered correctly, and the results are expressed in terms of a 100‐point scale.

### 2.3 Ethical considerations

This research was implemented under the approval of the Research Ethics Committee of Saitama Medical University, Saitama Medical Center (No. 2021‐106) and supported by JSPS KAKENHI Grant Number JP21K13709. Participation was voluntary and information was collected anonymously after obtaining consent from each respondent by assuring confidentiality throughout the data collection period.

### 2.4 Data analysis

We conducted the statistical analysis on SPSS Ver. 25 (IBM Corp.). Descriptive data include mean, standard deviation, range, and percentage values.

We examined four types of the following associations by Spearman's rank correlation, respectively, after confirming the normality of the data in advance:

1. The association between the Epilepsy Self‐Stigma Scale and the Knowledge Scale. The significance probability was set at *P* < .05;
2. The association between the three factors of the Epilepsy Self‐Stigma Scale (i.e., Internalization of stigma, Societal incomprehension, and confidentiality) and the Knowledge Scale. The significance probability was set at *P* < .05;
3. The association among the Rosenberg Self‐Esteem Scale, the epilepsy self‐stigma scale (the significance probability was set at *P* < .01), and the knowledge scale (the significance probability was set at *P* < .05);
4. the association among the seizure frequency, the epilepsy Self‐Stigma Scale, and the Rosenberg Self‐Esteem Scale. The significance probability was set at *P* < .01.

In addition, we analyzed descriptive statistics performed on the responses regarding the restrictions and difficulties due to epilepsy, desire for epilepsy support, and participation in self‐help groups and educational lectures. One of Japan's primary self‐help associations for epilepsy patients is the Japan Epilepsy Association. There are chapters in all 47 prefectures of Japan, and they plan, manage, and support various activities such as study groups and exchange meetings.^22^ In this survey, we asked about participation in the “Nami no kai” run by the Japan Epilepsy Association, and for those who do not participate, the reasons why they do not participate.

## 3 RESULTS

### 3.1 Participants' characteristics

We received the responses to the questionnaires from 310 participants. (mean age of 47.8 ± 11.9 years, 190 males and 120 females).

Other attributes and scale scores are shown in Table 1. Of the 310 respondents, 12 (3.9%) were aware that their seizures had been more frequent in the past 6 months, 223 (71.9%) said they had not changed, and 75 (24.2%) said they had improved their health conditions. Of the 310 respondents, 41 (13.23%) had an aura before the seizure, 44 (14.2%) had seizures that caused loss of consciousness, 33 (10.6%) had seizures with generalized convulsions, 19 (6.1%) had seizures that caused wandering or aimless movements, and 218 (70.3%) was unclear what type of seizures they had. Antiseizure medications were taken by 303 (97.7%) of 310 patients. Regarding perceived side effects, 185 of the 303 (61.1%) reported no side effects at all, 57 (18.8%) had side effects that did not bother them, 53 (17.5%) had tolerable side effects, and 8 (2.6%) had intolerable side effects.

**TABLE 1:** Participant characteristics and ESSS, RSES, EKS scores(N = 310)

Variables	Mean (SD)
Age, y	47.80 (11.91)
Range	21–77
Gender	
Female	38.7 (%)
Male	61.3 (%)
Length of education	14.07 (2.07)
Employment/life status	
Employed	58.7 (%)
Self employed	1.30%
On leave	6.1 (%)
Unemployed	28.4 (%)
Pensioners	0.6 (%)
Homemaker	2.3 (%)
Work transition training	0.3 (%)
Students	2.3 (%)
Living style	
Single	64 (%)
With family or partners	73.9 (%)
Group home	0.3 (%)
Seizure frequency	
No seizures in the last 6 mo	218 (70.3%)
1–2 seizures in the last 6 mo	35 (11.3%)
3–5 seizures in the last 6 mo	20 (6.5%)
At least once a month	18 (5.8%)
At least once a week	11 (3.5%)
At least once a day	8 (2.6%)
ESSS	17.69 (6.25)
1. Internalization of stigma	7.96 (3.26)
2. Societal incomprehension	5.24 (1.85)
3. Confidentiality	4.48 (2.11)
RSES	26.02 (6.13)
EKS	40.20 (18.75)

Abbreviations: EKS, Epilepsy Knowledge Scale; ESSS, Epilepsy Self‐Stigma Scale; RSES, Rosenberg Self‐Esteem Scale; SD, standard deviation.

### 3.2 Correlations between self‐stigma and knowledge

We used Spearman's rank correlation coefficient to examine the association between the Epilepsy Self‐Stigma Scale and the Knowledge Scale after a priori checking the normality of the data. Results showed no significant correlation between the epilepsy self‐stigma scale and the knowledge scale (*P* = .562). In addition, we used the three factors of the Epilepsy Self‐Stigma Scale (Internalization of stigma, societal incomprehension, and confidentiality) to examine the association between the three factors and knowledge after confirming the normality of the data a priori. Results showed no significant correlation between the three factors of the Epilepsy Self‐Stigma Scale and the Knowledge Scale (*P* = .416‐.716). We listed detailed values in Table 2.

**TABLE 2:** Correlations between ESSS (total score and 3 factors) and EKS

	EKS	
	*ρ*	*P*
ESSS	−.472	.56^*^
1: Internalization of stigma	−.52	.72^*^
2: Societal incomprehension	−.30	.46^*^
3: Confidentiality	−.051	.42^*^

Abbreviations: EKS, Epilepsy Knowledge Scale; ESSS, Epilepsy Self‐Stigma Scale.

^*^ *P* < .05.

### 3.3 Correlations among self‐stigma, self‐esteem, and epilepsy knowledge

The association among self‐esteem (RSES) and self‐stigma (ESSS) and knowledge (EKS), after confirming the normality of the data a priori. The results showed a significant moderate negative correlation between self‐esteem and self‐stigma (*P* < .001, *ρ* = −.423). A significant weak positive correlation was found between self‐esteem and knowledge (*P* = .005, *ρ* = .177). Detailed values are shown in Table 3.

**TABLE 3:** Correlations among RSES scores and ESSS and EKS (N = 310)

	ESSS	EKS
RSES		
Spearman's rank correlation coefficient	−0.423*	0.18**
	.40 < *ρ* < .70	*ρ* < .40

Abbreviations: EKS, Epilepsy Knonwledge Scale; ESSS, Epilepsy Self‐Stigma Scale; RSES, Rosenberg Self‐Esteem Scale.

* *P* < .001

** *P* = .005.

### 3.4 Correlations among seizure frequency, self‐stigma, and self‐esteem

The association among seizure frequency, self‐stigma (ESSS), and self‐esteem (RSES), after confirming the normality of the data a priori. The results showed a significant weak negative correlation between self‐stigma and seizure frequency (*P* < .001, *ρ* = −.162). Results showed no significant correlation between seizure frequency and self‐esteem (*P* = .224).

### 3.5 Descriptive analysis of restrictions and difficulties due to epilepsy

We asked the participants, “How would you emotionally assess the extent of your life feeling restricted or constrained due to your ‘epilepsy’ in the last 6 months?” 98 (31.6%) of 310 said “was not restricted at all,” 83 (26.8%) said “was limitedly restricted,” and 71 (22.9%) responded “some extent,” 37 (11.9%) responded “considerably,” and 21 (6.8%) “extremely,” respectively. The results of this question are shown in Table 4. The item that most constrained the respondents based on the result was riding a vehicle, and 18.4% said they were extremely restricted. On the contrary, the item that constrained the respondents the least was the relationship with their partners. A modest 3.9% answered that they were extremely constrained, while 70.3% of the respondents felt no constraints from epilepsy.

**TABLE 4:** How would you emotionally assess the extent of your life feeling restricted or constrained due to your “epilepsy” in the last 6 mo? (N = 310)

Restricted or restricted content	Extremely	Considerably	Some extent	limitedly	Not at all
Working and studying.	17	16	53	60	164
	5.5%	5.2%	17.1%	19.4%	52.9%
Going out without a chaperone.	15	14	41	33	207
	4.8%	4.5%	13.2%	10.6%	66.8%
**By driving a bicycle, motorcycle, or car** *	57	26	39	46	142
	**18.4%**	8.4%	12.6%	14.8%	45.8%
By using trains, buses, or other transportation	15	16	40	27	212
	4.8%	5.2%	12.9%	8.7%	68.4%
By sports, leisure time, hobbies, etc.	15	18	42	44	191
	4.8%	5.8%	13.5%	14.2%	61. 6%
Living alone	18	19	52	47	174
	5.8%	6.1%	16.8%	15.2%	56.1%
Relationships with friends	14	15	39	38	204
	4.5%	4.8%	12.6%	12.3%	65.8%
**Relationship with partner** **	12	10	36	34	218
	**3.9%**	3.2%	11.6%	11.0%	**70.3%**
Family relations	16	15	43	51	185
	5.2%	4.8%	13.9%	16.5%	59.7%
Financial matters	33	26	63	41	147
	10.6%	8.4%	20.3%	13.2%	47.4%
Memory (forgetfulness)	31	24	55	57	143
	10.0%	7.7%	17.7%	18.4%	46.1%
Physical functioning	20	24	53	49	164
	6.5%	7.7%	17.1%	15.8%	52.9%
Health	26	28	60	60	136
	8.4%	9.0%	19.4%	19.4%	43.9%
Mood	28	37	57	66	122
	9.0%	11.9%	18.4%	21.3%	39.4%
**Through the above, how restricted do you feel because of your epilepsy?**	**21**	**37**	**71**	**83**	**98**
	**6.8%**	**11.9%**	**22.9%**	**26.8%**	**31.6%**

^*^ The item most constraining to respondents.

^**^ The item least constraining to respondents.

As for the question “if having epilepsy caused any problems in school or work,” 35 (18.8%) of the 186 patients who are in occupation or education responded “frequently,” 69 (37.1%) responded “sometimes,” and 82 (44.1%) responded “nothing.” Also, 66 (35.5%) of the people who answered said that their epilepsy kept them from doing some things at school or work, like going to the pool, working the night shift, and driving a car. In addition, when asked if the respondents had ever experienced any discrimination from their employers, coworkers, teachers, or classmates due to epilepsy, 91 (49%) participants answered they had no such experience. Twenty‐nine (15.6%) participants claimed they had experienced discrimination, while 61 (19.7%) indicated they never disclosed their medical records to others.

### 3.6 Descriptive analysis of desire for support for epilepsy and participation in self‐help groups and educational lectures

When asked by whom they most often seek help for epilepsy in this survey, 130 respondents said doctors, 125 respondents followed parents, and 112 respondents followed their partners. Five respondents said they sought help from self‐help associations, and 40 said they had no one to help with their epilepsy. Fifteen who answered “other” included searching online, visiting care services, and visiting nurses. The details are shown in Table 5.

**TABLE 5:** Who do you often ask for help with your epilepsy? (Multiple answers allowed question) (N = 310)

	Number	%
Partner	112	36.1
Parents	125	40.3
Siblings	49	15.8
Child	29	9.4
Friend	18	5.8
Self‐help group (patient group)	5	1.6
Physician	131	42.3
Colleague at work	24	7.7
Others	15	4.8
I have no one to ask for help	40	12.9

Of the 270 respondents who reported receiving this aid, 57 (21.1%) were highly satisfied with this assistance, 98 (36.3%) were satisfied, 99 (36.7%) were fair, and 1 (36.7%) were not highly satisfied. Eleven (4.1%) and 5 (1.9%) said they were not satisfied at all.

In our survey, when asked if they were involved in an epilepsy self‐help group such as “Nami no Kai” (as the Japan Epilepsy Association), 18 (5.8%) answered yes, and 292 (94.2%) said they had never been involved. Regarding participation in educational courses and lectures on epilepsy, we also distributed this question to18 respondents (5.8%) who participated in such events. As for specific reasons for no experience in such educational programs, the most common reason given by 36 respondents was that they did not know such programs existed in the first place. Other reasons included lack of time or money, unwillingness to reveal their health conditions to the public, resistance to attending such programs due to potential prejudice, possible damage to their self‐esteem, etc.

## 4 DISCUSSION

### 4.1 Factors associated with self‐stigma in Japanese patients with epilepsy

Our results showed a significant moderate negative correlation between self‐esteem and self‐stigma in patients with epilepsy (*P* < .001, *ρ* = −.423). A significant weak positive correlation was found between self‐esteem and knowledge (*P* = .005, *ρ* = .177). These results suggest that sufficient knowledge of epilepsy may improve the self‐esteem of patients with epilepsy and thus reduce the self‐stigma associated with epilepsy.

Previous reports have described an association between high seizure frequency and high stigma of epilepsy.^7^, ^9^, ^11^, ^12^, ^13^ However, the results of our study showed a negative and weak correlation between seizure frequency and self‐stigma (*P* < .001, *ρ* = −.162). Our results suggested that long‐term life experiences are more likely to cause self‐stigma than the frequency of seizures in the past 6 months. This is consistent with reports that one‐fifth of seizure‐free patients continue to feel stigma even after being seizure‐free for more than 2 years. Improving seizure treatment status alone is not enough to reduce stigma.^23^ In our research, 91 (49%) of 186 participants had experienced any discrimination from their employers, coworkers, teachers, or classmates due to epilepsy.

These results suggest that short‐term epilepsy treatment for seizure control is insufficient to reduce self‐stigma in patients with epilepsy. The dissemination for people to enable sufficient epilepsy knowledge and positive perceptions of epilepsy by increasing self‐efficacy throughout a lifetime may reduce self‐stigma.

### 4.2 Knowledge of patients with epilepsy and the potential for self‐help groups and educational programs

Prospective cohort studies in Europe have shown that people with epilepsy have low grades and educational attainment.^24^ In our research, the mean number of years of education was 14.07 ± 2.07 years. Considering that compulsory education in Japan is 12 years and the average schooling in Japan is 12.8 years, the educational level of the participants in this survey is not low. However, the knowledge means score results showed 40.20 ± 18.75 points. This is lower than the average score of 48.50 ± 19.39 points in a survey from Germany,^20^ where this knowledge scale was developed. This suggests that the sufficient knowledge of epilepsy in Japan has not yet been disseminated.

A psychosocial education program for patients with epilepsy has shown improvements in knowledge.^25^ In contrast, the participation rate of patients in self‐help groups and educational lectures was at a modest 5.8% in this survey. The low participation rate in these programs may be due not only to the lack of awareness but also to the concentration of facilities and locations in the greater Tokyo area. Since educational programs on specialized diseases are often held predominantly at epilepsy centers, it may be difficult for patients living in rural areas to access them. Japan is an archipelago nation, and the foremost hospitals and university hospitals that provide specialized medical care, not only for epilepsy, are concentrated in urban areas. This study was implemented online. Similarly, online educational programs provided through smartphones and other communication devices could be used to improve knowledge regardless of accessibility and the living environment.

In addition, 61 (19.7%) of the respondents indicated they never disclosed their medical records to others, and they also gave reasons for not participating in self‐help groups, unwillingness to reveal their health conditions to the public, resistance to attending such programs due to potential prejudice, possible damage to their self‐esteem. Self‐stigma prevents people from telling others about their illness, as 49% of respondents have experienced discrimination due to epilepsy. It is anticipated that if knowledge can be provided through online and other methods to reduce self‐stigma, participation in self‐help groups will be easier.

### 4.3 Limitation

This study is limited because it was conducted among patients registered with an online survey service. Not all patients with epilepsy in Japan participated in our study, focusing on a generation with a high affinity for the Internet. In addition, the survey was based on self‐assessment and did not require diagnosed types of epilepsy or an otherworldly assessment of seizure frequency from medical records. Detailed information from existing medical records would be needed to investigate the association between seizure frequency, stigma, and other factors. We should note that the present findings cannot be generalized to all individuals with epilepsy in modern Japanese society.

## 5 CONCLUSIONS

Our research identifies previously unknown factors associated with self‐stigma among Japanese patients with epilepsy. There was a significant moderate negative correlation between self‐esteem and self‐stigma and a significant weak negative correlation between seizure frequency and self‐stigma. Japanese patients with epilepsy feel a sense of self‐stigma even when their seizures are under control. Even though controlling seizures is an important part of treating epilepsy, we must take a long‐term view and work to improve the self‐esteem of people with epilepsy. In addition, we discovered a significant weak positive correlation between self‐esteem and knowledge. To reduce the self‐stigma of patients with epilepsy, we consider it essential to provide interventions that assist people with epilepsy in acquiring accurate knowledge about epilepsy and in enhancing their long‐term self‐esteem. Regarding involvement in self‐help groups, educational lectures, and psychosocial education programs, which are said to be beneficial for enhancing epilepsy knowledge, we discovered that self‐stigma inhibits participation in Japan. We believe that, in the future, it will be essential to develop educational programs that are more accessible to those with self‐stigma.

## CONFLICT OF INTEREST

None of the authors has any conflict of interest to disclose.

## ETHICAL APPROVAL

The study was conducted after the approval of the study protocol by the institutional review board of Saitama Medical Center, Saitama Medical University (approval no. 2021‐106). Participation was voluntary, and information was collected anonymously after obtaining written consent from each respondent. Participants were assured that their data would be kept confidential throughout the data collection period. We confirm that we have read the Journal's position on issues involved in ethical publication and affirm that this report is consistent with those guidelines.

## Data Availability

The study data are not publicly available.
